# Bromodomain and Extra-Terminal Protein Inhibition Attenuates Neutrophil-dominant Allergic Airway Disease

**DOI:** 10.1038/srep43139

**Published:** 2017-02-24

**Authors:** Michelle L. Manni, Sivanarayana Mandalapu, Andres Salmeron, Jose M. Lora, Jay K. Kolls, John F. Alcorn

**Affiliations:** 1 Department of Pediatrics, Children’s Hospital of Pittsburgh of UPMC, Pittsburgh, PA USA; 2 Constellation Pharmaceuticals, Inc., Cambridge, MA USA; 3 Richard King Mellon Foundation Institute for Pediatric Research, Children’s Hospital of Pittsburgh of UPMC, Pittsburgh, PA USA

## Abstract

Atopic asthma is a prevalent respiratory disease that is characterized by inflammation, mucus hypersecretion, and airway hyperresponsiveness. The complexity of this heterogeneous disorder has commanded the need to better define asthma phenotypes based on underlying molecular mechanisms of disease. Although classically viewed as a type 2-regulated disease, type 17 helper T (Th17) cells are known to be influential in asthma pathogenesis, predominantly in asthmatics with neutrophilia and severe refractory disease. Bromodomain and extra-terminal domain (BET) chromatin adaptors serve as immunomodulators by directly regulating Th17 responses and Th17-mediated pathology in murine models of autoimmunity and infection. Based on this, we hypothesized that BET proteins may also play an essential role in neutrophil-dominant allergic airway disease. Using a murine model of neutrophil-dominant allergic airway disease, we demonstrate that BET inhibition limits pulmonary inflammation and alters the Th17-related inflammatory milieu in the lungs. In addition, inhibition of BET proteins improved lung function (specifically quasi-static lung compliance and tissue elastance) and reduced mucus production in airways. Overall, these studies show that BET proteins may have a critical role in asthma pathogenesis by altering type 17 inflammation, and thus interfering with BET-dependent chromatin signaling may provide clinical benefits to patients suffering from asthma.

Asthma is a chronic inflammatory disease of the airways and represents a significant health burden worldwide^1^,^2^. Allergen-specific CD4^+^ T helper (Th) cells drive atopic asthma pathogenesis, which is characterized by pulmonary inflammation, mucus cell metaplasia, airway remodeling, and airway hyperresponsiveness (AHR). Although once viewed as a predominantly Th2 cell-dominant disorder, it is now recognized that asthma is a clinically heterogeneous disease. In severe refractory asthma, Th2 and/or Th17 cells are thought to contribute to disease pathogenesis. Specifically, Th17 cells, which produce IL-17 and IL-22, are known to mediate neutrophilia and influence asthma severity^3^,^4^,^5^,^6^. Our recent work has recognized this Th17-related phenotype in asthmatics with severe refractory disease, and has shown that different molecular mechanisms likely contribute to each of the observed asthma pathologies (inflammation, AHR, and mucus production)^7^. Although Th2-related disease is well studied, the complexity and diversity of the clinical presentation of asthma warrants further study to elucidate and clarify the molecular mechanisms underlying disease and ultimately establish more effective therapies.

Acetylation of histones promotes the recruitment of effector proteins, alters the confirmation of chromatin, and regulates transcription of inflammatory and immunoregulatory genes^8^. Bromodomain and extra-terminal (BET) proteins possess bromodomain motifs that bind acetylated lysine residues in histones, thereby regulating transcription of genes involved in cellular proliferation, cell cycle progression and apoptosis^9^. The BET family of adaptor proteins is composed of Brd2, Brd3, Brd4, and testis-specific Brdt. Selective, small molecule inhibitors of BET proteins displace BET bromodomains from chromatin by competitively binding to the aceyl-lysine recognition pocket. This disruption of the interaction between BET proteins and acetylated histones has been shown to impede transcription of genes that mediate cellular growth and survival in cancers^9^,^10^,^11^,^12^,^13^,^14^, control inflammatory responses^15^, and regulate cell function of innate and adaptive immune cells^16^,^17^,^18^,^19^.

Based on the fundamental role of BET bromodomains in cell growth and proliferation, recent studies have focused on establishing a link between BET proteins and inflammatory responses in disease. Mele and colleagues have shown that BET proteins are critical for controlling both human and murine Th17 differentiation and activation as well as for regulating Th17-associated cytokine production (IL-17, IL-21, and GM-CSF)^16^. More importantly, their work showed that the BET family members Brd2 and Brd4 critically modulate Th17 biology by associating with the *Il17* locus in a bromodomain-dependent manner, thereby directly controlling the transcription of IL-17^16^. A study by Chen *et al*. also showed BET inhibition repressed Th17 cell responses in explanted tissues from cystic fibrosis patients^20^. Aside from circulating immune cells, BET proteins have been shown to influence cell function and response of airway epithelial cells^20^,^21^, airway smooth muscle^22^,^23^, and lung fibroblasts^24^. Further, pharmacological inhibition of BET bromodomains was protective in murine models of autoimmunity^16^, bleomycin-induced pulmonary fibrosis^24^, LPS-induced shock^18^, and *Pseudomonas aeruginosa* lung infection^20^.

Although the role of epigenetic regulation in asthma pathogenesis remains unclear, evidence of altered histone acetylation, aberrant histone acetyltransferases and histone deacetylases expression, as well as abnormal expression of genes involved in pulmonary repair and inflammation has been reported in the airways of asthmatics^21^,^22^,^23^,^25^,^26^,^27^,^28^,^29^. Additionally, mimics of BET bromodomains have been shown to inhibit airway smooth muscle cell proliferation and cytokine release from patients with asthma^22^,^23^. Based on these findings and the role of BET proteins in Th17 cell function, we hypothesize that the BET inhibitor CPI-203 would limit asthma pathogenesis in a Th17-induced murine model of severe refractory asthma. Overall, this work establishes a role for BET proteins in Th17-mediated allergic airway disease and suggests that interfering with BET-dependent chromatin signaling may provide clinical benefits to patients suffering from asthma.

## Materials and Methods

### Mice

6–8 week old, female BALB/c SCID and DO11.10 TCR-transgenic mice were purchased from Taconic. All mice were housed in a pathogen-free environment at the Children’s Hospital of Pittsburgh of UPMC and were given food and water ad libitum. All animal experiments were reviewed and approved by the University of Pittsburgh Institutional Animal Care and Use Committee. All experiments were performed in accordance with IACUC guidelines and regulations.

### *In vitro* differentiation of Th17 cells

CD4^+^ CD62L^+^ naïve T cells from the spleens of DO11.10 TCR-transgenic mice were enriched using a CD4^+^ CD62L^+^ T cell Isolation Kit II (Miltenyi Biotec) and were cultured for 6 days with antiCD3/antiCD28 mouse Dynabeads (Invitrogen) under Th17 cell polarizing conditions (10 ng/mL IL-23, 1 ng/mL TGFβ, 2 ng/mL IL-6, 10 μg/mL anti-IL-4, and 10 μg/mL anti-IFNγ purchased from R&D Systems) as previously described^3^.

### Th17-induced, neutrophil-dominant allergic airway disease model

BALB/c SCID mice were challenged with 50 μg of ovalbumin (Sigma-Aldrich) via oropharyngeal aspiration (OA) on Day 0. On Day 1, 1 × 10^6^ Th17 cells were adoptively transferred by retro-orbital injection. Mice were subsequently challenged with 50 μg ovalbumin OA daily for three consecutive days after cell transfer (Days 2–4). Twenty-four hours after the last challenge, mice were euthanized (Day 5) and allergic airway disease was assessed as detailed below. Control mice received all ovalbumin challenges, but received phosphate buffered saline retro-orbitally at time of cell transfer. To inhibit BET bromodomains, mice were treated with 2.5 mg/kg BET inhibitor CPI-203 (Constellation Pharmaceuticals) via intraperitoneal injection twice daily on Days 1–4.

### Lung Mechanics Measurements

Pulmonary function was assessed by mechanical ventilation of anesthetized (90 mg/kg pentobarbital-NA, IP) and tracheotomized mice using a computer-controlled small-animal mechanical ventilator (FlexiVent; SCIREQ) as previously described^30^,^31^. Respiratory mechanics measurements were made prior to and following inhalation of aerosolized methacholine (0, 0.75, 3.125, 12.5, and 25 mg/mL).

### Bronchoalveolar lavage (BAL) and lung processing

After lung mechanics analyses, BAL fluid was collected via the intratracheal instillation and recovery of 1 mL of PBS. Total cells in the recovered BAL fluid were counted using a hemocytometer and the number of neutrophils, eosinophils, macrophages and lymphocytes were quantified from modified Wright-Giemsa-stained cytospin preparations of BAL cells (200 cells/slide). Lung lobes were separated and processed as follows: flash frozen in liquid nitrogen for cytokine and chemokine analyses by Lincoplex according to manufacturer’s instructions (Bio-Plex Pro™ Mouse Cytokine Group I Panel 23-Plex, BioRad) or gene expression analyses using quantitative RT-PCR, or inflation-fixed with 10% buffered formalin and paraffin embedded for histology^32^,^33^. For Lincoplex analyses, the following cytokines and chemokines were measured (the limit of detection): IL-1α (2 pg/mL), IL-1β (7 pg/mL), IL-2 (3 pg/mL), IL-3 (2 pg/mL), IL-4 (3 pg/mL), IL-5 (2 pg/mL), IL-6 (2 pg/mL), IL-9 (15 pg/mL), IL-10 (2 pg/mL), IL-12p40 (2 pg/mL), IL-12p70 (4 pg/mL), IL-13 (9 pg/mL), IL-17A (1 pg/mL), EOTAXIN (148 pg/mL), G-CSF (1 pg/mL), GM-CSF (7 pg/mL), IFNγ 6 pg/mL), CXCL1 (3 pg/mL), MCP-1 (14 pg/mL), MIP-1α (24 pg/mL), MIP-1β (2 pg/mL), CCL5 (5 pg/mL), TNFα (6 pg/mL).

### Gene expression analysis

RNA was isolated from lung tissue using an Absolutely RNA Miniprep Kit (Agilent Technologies, Santa Clara, CA). One μg of RNA was then converted to cDNA using iScript cDNA Synthesis Kit (Bio-Rad, Hercules, CA). Gene expression analysis was performed using Taqman Fast Mastermix and Assay On Demand TaqMan primer and probe sets for the following murine genes of interest: Muc5ac, Muc5b, Clca3, Spp1 (Life Technologies, Grand Island, NY).

### Histological Scoring of Inflammation and Mucus Metaplasia

To characterize tissue inflammation in the lung, standard H&E-stained lung sections were scored by observers (JFA and MLM), who were blinded to the sample group identity. The entire lung section was observed with a light microscope (×40 magnification) and peribronchial, perivascular, and parenchymal inflammation were scored as previously described^7^,^31^. Briefly, cellular inflammation present in each area was scored according to the following scale: 0 = no inflammation, 1 = up to 25%, 2 = 25–50%, 3 = 50–75%, and 4 = 75–100%. The inflammation score was reported as the mean of the scores for each sample group. Mucus production was quantified through the observation of Periodic Acid-Schiff (PAS) stained lung sections by observers (JFA and MLM) blinded to the identity of the sample groups. The entire lung section was observed with a light microscope (100x magnification) and the amount of PAS staining in the entire lung section was scored using the following scale: 0 = no staining present, 1 = light staining in large airway only, 2 = staining present in all large airways and some small bronchioles, 3 = staining present in large and small airways, and 4= dense staining in all airways. The PAS score was reported as the mean of the scores for each sample group.

### Statistical analyses

Data were analyzed using GraphPad Prism 5.0 (GraphPad Software Inc., La Jolla, CA). Experiments involving 2 variables were analyzed by two-way analysis of variance with a Bonferonni post-hoc test. Data with one variable were analyzed using one-way analysis of variance with Tukey’s post-hoc test. Data comparing two groups were analyzed using an unpaired t-test. Data was log transformed for statistical analyses where indicated. Data shown are mean ± SEM. A value of p < 0.05 was considered to be statistically significant.

## Results

### Inhibition of BET proteins decreases pulmonary inflammation and alters cytokine and chemokine production in the lungs during Th17-induced allergic airway disease

**Figure 1:**
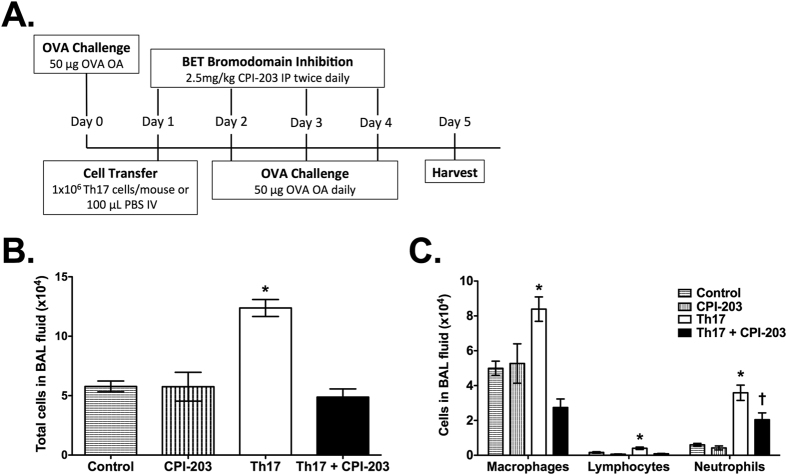
BET bromodomain inhibition reduces Th17-induced airspace inflammation in the lungs during allergic airway disease in mice. (**A**) BALB/c SCID mice were treated with ovalbumin (OVA) and adoptively transferred with Th17 cells to induce allergic airway disease and the therapeutic potential of CPI-203 was investigated in this model. Cellular inflammation in the airspaces was measured by (**B**) total cells in the BAL fluid and (**C**) BAL fluid cell differential counts. Mice in control and CPI-203 groups received all OVA challenges, but phosphate buffered saline retro-orbitally at time of cell transfer. Graphs show data for control (n = 5), CPI-203 (n = 6), Th17 (n = 8), Th17+ CPI-203 (n = 8) combined from three independent experiments. *p < 0.05 when compared to all other groups, †p < 0.05 when compared to the CPI-203 group.

Previous work has shown that adoptive transfer of OVA-specific Th17 cells into OVA-treated BALB/c SCID mice is sufficient to promote neutrophilic inflammation, AHR, and mucus metaplasia, which are not affected by glucocorticoid treatment^3^. Utilizing this model, we investigated the therapeutic potential of CPI-203, a highly selective and potent inhibitor of BET proteins (Fig. 1A). Similar to previous work^3^,^7^, adoptive transfer of OVA-specific Th17 cells into OVA-treated BALB/c SCID mice increased cellular inflammation in the airspaces, resulting in higher levels of macrophages, neutrophils, and lymphocytes (Fig. 1B,C). CPI-203 treatment during Th17-driven allergic airway disease attenuated inflammation in the airspaces of the lungs and resulted in significantly lower levels of macrophages, lymphocytes, and neutrophils (Fig. 1B,C). Overall, these results suggest that the steroid-insensitive neutrophilia in the airspaces that was induced by Th17-mediated allergic airway disease, was reduced by CPI-203 treatment.

**Figure 2:**
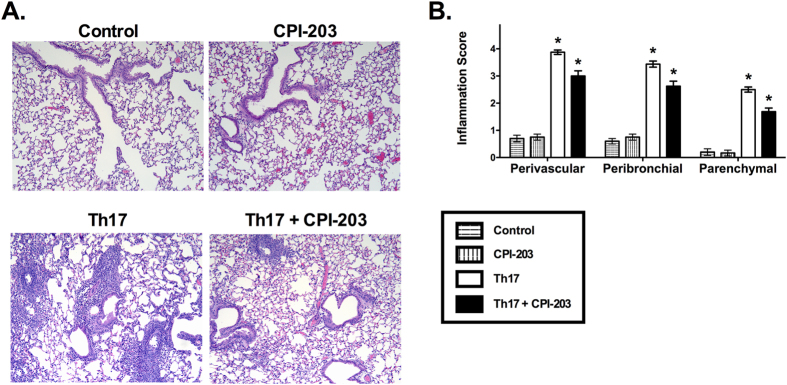
Inhibition of BET protein and chromatin interactions alters tissue-associated inflammation in the lungs of mice with Th17-induced allergic airway disease. (**A**) Representative images of hematoxylin and eosin-stained lung sections (100x magnification) from OVA-treated BALB/c SCID mice that received Th17 cells (Th17 group) or PBS (control group) with and without CPI-203 treatment. (**B**) Perivascular, peribronchial, and parenchymal-associated inflammation in the entire lung section were quantified by an observer blinded to the identity of the samples. Graph shows data for control (n = 5), CPI-203 (n = 6), Th17 (n = 8), Th17+ CPI-203 (n = 8) combined from three independent experiments. *p < 0.05 when compared to all other groups, †p < 0.05 when compared to the CPI-203 group.

In addition to reducing cellular inflammation present in the airspaces, inhibition of BET proteins during Th17-induced allergic airway disease also decreased inflammation present in the lung tissue. Histological assessment of hematoxylin and eosin-stained lung sections showed that perivascular, peribronchial, and parenchymal inflammation were all lessened by CPI-203 treatment in this murine model of Th17-induced allergic airway disease (Fig. 2). Furthermore, treatment with CPI-203 altered cytokine and chemokine levels in the lungs of Th17 adoptive transfer, OVA challenged mice. Specifically, IL-1α, IL-1β, IL-2, IL-6, IL-10, IL-12p40, IL-12p70, IL-13, IL-17A, and eotaxin increased in response to CPI-203 treatment, while G-CSF, CXCL1, MIP-1β, and CCL5 decreased (Table 1). Several cytokines and chemokines were also unaffected by CPI-203 treatment, which included IL-3, IL-4, IL-5, IL-9, GM-CSF, IFNγ, MCP-1, MIP-1α and TNFα. These data demonstrate that inhibition of BET proteins alters Th17 cell-driven inflammation by modulating downstream pro-inflammatory mediators, which promote neutrophil recruitment into the lungs.

### BET bromodomain inhibition with CPI-203 alters respiratory mechanics in mice with Th17-induced allergic airway disease

**Figure 3:**
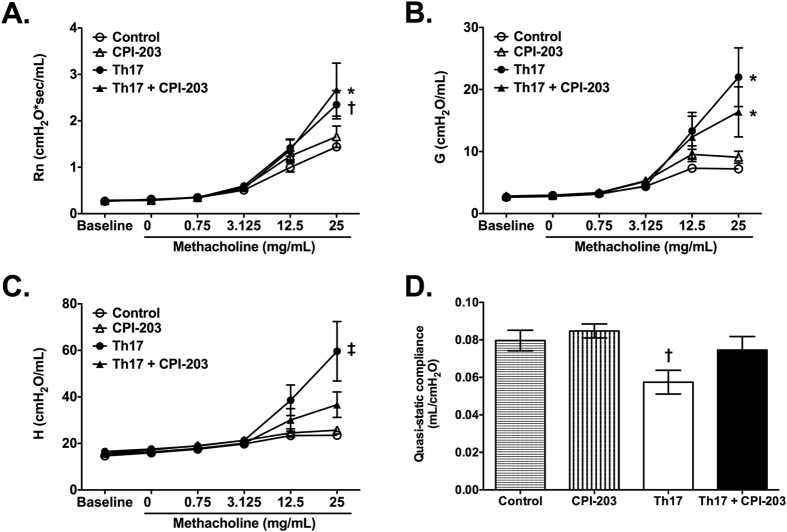
Blocking BET protein chromatin signaling via CPI-203 treatment alters tissue elastance in the lungs of mice with Th17-induced allergic airway disease. Lung function was evaluated following CPI-203 treatment in a model of Th17-induced allergic airway disease. (**A–C**) Airway responsiveness to inhaled aerosolized methacholine (Newtonian resistance (Rn), tissue damping (**G**), and tissue elastance (**H**)), and (**D**) quasi-static lung compliance were measured using a FlexiVent system. Graphs show data for control (n = 5), CPI-203 (n = 6), Th17 (n = 8), Th17 + CPI-203 (n = 8) combined from three independent experiments. *p < 0.05 when compared to control and CPI-203 group, †p < 0.05 when compared to the control group, ‡p < 0.05 when compared to all other groups.

To further characterize the role of BET proteins in Th17-dominant allergic airway disease, lung function was assessed by measuring Newtonian resistance (Rn), tissue damping (G), and tissue elastance (H), in response to increasing doses of methacholine as well as by measuring quasi-static lung compliance. Inhibition of BET proteins with CPI-203 treatment did not affect Rn, a parameter that is representative of central or conducting airway resistance, and G, a parameter that relates to tissue or parenchymal resistance, in this model of Th17-driven allergic airway disease (Fig. 3A,B). However, blocking BET bromodomains significantly lowered tissue elastance (H), a parameter related to parenchymal recoil, in Th17 cell adoptive transfer, OVA challenge mice (Fig. 3C). CPI-203 treatment also trended to improve quasi-static lung compliance in mice with Th17-induced allergic airway disease (Fig. 3D).

### Inhibition of BET bromodomains limits Th17-mediated mucus production in the lungs of mice during allergic airway disease

**Figure 4:**
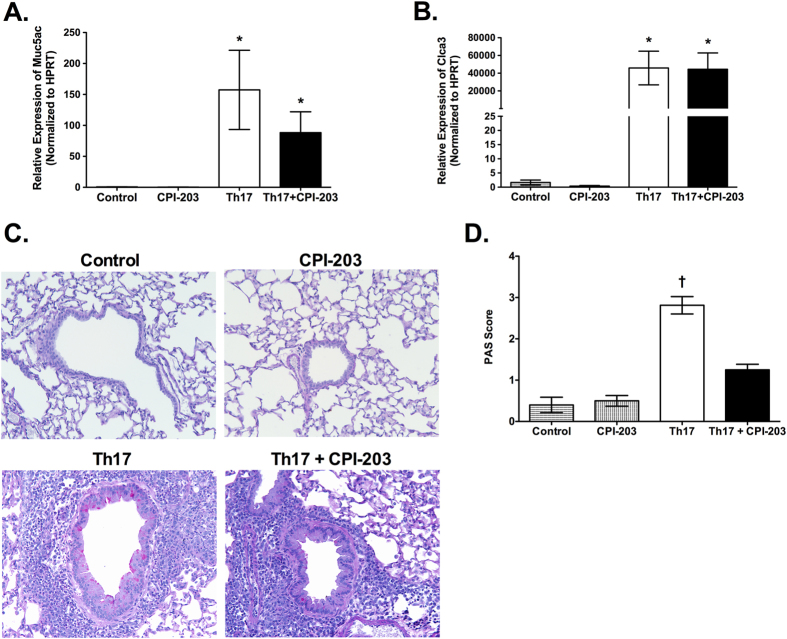
BET bromodomain inhibition via CPI-203 treatment alters goblet cell hyperplasia and mucus production in the lungs of mice with Th17-induced allergic airway disease. (**A**) Pulmonary expression of *Muc5ac* and (**B**) *Clca3* as well as (**C**) representative images of Periodic Acid Schiff (PAS)-stained lung sections (200x magnification) from OVA-treated BALB/c SCID mice that received Th17 cells (Th17 group) or PBS (control group) with and without CPI-203 treatment. (**D**) Mucus production in the PAS-stained lung sections was quantified by an observer blinded to the identity of the samples. Data shown for control (n = 5), CPI-203 (n = 6), Th17 (n = 8), Th17+ CPI-203 (n = 8) combined from three independent experiments. *p < 0.05 when compared to control and CPI-203 group, †p < 0.05 when compared to all other groups.

As previously reported^3^, adoptive transfer of Th17 cells into OVA-treated BALB/c SCID mice increased pulmonary mRNA expression of mucus-associated genes, *Muc5ac* and *Clca3*, and increased mucus production as evident by PAS staining present in the airways (Fig. 4). In this study, inhibition of BET bromodomains during Th17-induced allergic airway disease significantly reduced PAS-positive airways, while not significantly altering *Muc5ac* and *Clca3* mRNA expression (Fig. 4). These results suggest that BET proteins are important drivers of mucus production in the airways during allergic airway disease, independent of promoting mucin gene expression.

## Discussion

In this study, we show that BET inhibition limits neutrophilia and Th17-driven cytokine and chemokine release in the lungs in a murine model of severe, steroid-insensitive asthma. Inhibition of BET proteins also improved lung function, specifically quasi-static lung compliance and tissue-related AHR, and reduced mucus production in the airways. To our knowledge, these results are novel and demonstrate that BET proteins are important regulators of asthma pathogenesis, and more significantly can affect severe, steroid-insensitive disease. Therefore, our work herein demonstrates that BET inhibitors may provide therapeutic benefit to patients with asthma, especially those with neutrophilia and/or severe refractory disease.

Previous work has suggested that cellular infiltration into the airspaces during Th17-induced allergic airway disease correlated with quasi-static lung compliance, whereas AHR was associated with tissue-based inflammation^7^. The findings of this current study support these results, which demonstrate that when Th17-induced cellular inflammation is limited via BET protein inhibition, quasi-static lung compliance is also improved. Similarly, we also observe a marked reduction in Th17-induced tissue inflammation following BET protein inhibition and lower tissue-related AHR parameters. Although quasi-static lung compliance and tissue-related AHR may be associated with pulmonary inflammation, our results imply that central airway resistance is likely an inflammation-independent process. Alterations in airway resistance have been attributed to direct effects on airway remodeling and contractility. Specifically, IL-17 is known to induce airway smooth muscle cell contraction, thereby altering airway hyperresponsiveness^34^,^35^,^36^. Overall, these results highlight the complexity of the underlying biology that contributes to asthma pathologies. Further work is needed to establish how histone modifications affect tissue responses in asthma.

IL-17 is a cytokine mainly produced by Th17 cells and is thought to regulate neutrophilic inflammation and steroid resistance in severe asthmatics. BET chromatin modulators directly regulate Th17 responses and critically mediate Th17 biology via bromodomain-dependent association with acetylated histones at the *IL17* locus in Th17 cells^16^. Further, BET inhibition was shown to limit Th17-mediated autoimmunity and pulmonary infection^16^,^20^. Thus, we hypothesized that BET inhibition would alter Th17 cell-driven allergic airway disease. In this study, we did not observe a direct change in IL-17 protein following CPI-203 treatment, but did find lower levels of IL-17-driven cytokines and chemokines (G-CSF, CXCL1), neutrophils in the airspaces, and tissue inflammation in the lungs. The murine model of asthma utilized for this work is a neutrophil-dominant allergic airway disease model induced by antigen-specific Th17 cells, which presumably produce IL-17 as early as the first antigen challenge (Day 2, Fig. 1A). The lung tissue is collected for analyses several days after this initial challenge and thus may account for the unchanged pulmonary levels of IL-17 observed. The reduction of pulmonary neutrophilia and IL-17-driven cytokines implies however that the inhibition of BET proteins reduced the activity of IL-17 in the lungs in this murine model of severe, steroid-insensitive asthma.

Our data provides evidence that the inhibition of BET proteins lessens neutrophilic inflammation likely by altering downstream pro-inflammatory mediators, which promote neutrophil recruitment into the lungs. Other studies using BET mimics have also established that BET proteins can modulate neutrophilia by regulating IL-6 and CXCL8 release from human bronchial epithelial cells and airway smooth muscle cells from healthy controls and asthmatics^21^,^22^. BET protein function was also shown to be required for murine macrophage inflammatory processes^18^, which may influence their phenotype and role in asthma pathogenesis. Analyses of the cytokine and chemokine milieu following BET inhibition in mice with Th17 cell-driven allergic airway disease revealed a complex inflammatory environment even when cellular inflammation in the airspace and interstitium was significantly reduced. Overall, BET proteins clearly function as modulators of the inflammatory responses in this murine model of severe, steroid-insensitive asthma.

JQ1 and CPI-203, an analog of JQ1, are well-characterized BET bromodomain inhibitors that are extremely selective, with no off-target effects at biologically relevant or suprapharmalogical concentrations^37^,^38^,^39^. Clinical trials using BET bromodomain inhibitors for treatment of leukemias, lymphomas, myelomas, and other cancers are ongoing^40^. Recent studies have suggested the utility of BET mimics in Th17 cell-driven autoimmunity, pulmonary infection, and idiopathic pulmonary fibrosis^16^,^20^,^24^. Our original work herein demonstrates a pivotal role of BET proteins in asthma and more importantly, provides evidence that BET inhibition may be beneficial in a steroid-insensitive disease setting. As some asthmatics with severe glucocorticoid resistance show abnormal histone acetylation patterns^26^, pharmacological intervention targeting BET adaptor proteins may be efficacious to lessen disease burden for patients with refractory disease.

## Additional Information

**How to cite this article:** Manni, M. L. *et al*. Bromodomain and Extra-Terminal Protein Inhibition Attenuates Neutrophil-dominant Allergic Airway Disease. *Sci. Rep.* **7**, 43139; doi: 10.1038/srep43139 (2017).

**Publisher's note:** Springer Nature remains neutral with regard to jurisdictional claims in published maps and institutional affiliations.

## Figures and Tables

**Table 1:** Inflammatory cytokine and chemokine levels in the lungs following CPI-203 treatment in a murine model of Th17-induced allergic airway disease.

	Control (n = 5)	CPI-203 (n = 6)	Th17 (n = 8)	Th17+CPI-203 (n = 8)
**IL-1α**	22.57 ± 1.97	22.81 ± 1.00	142.75 ± 4.33*	166.47 ± 8.83*
**IL-1β**	233.89 ± 14.18	240.68 ± 5.64	2067.55 ± 37.30*	2415.43 ± 85.61*
**IL-2**	55.99 ± 4.25	58.81 ± 2.25	189.90 ± 7.53*	248.55 ± 13.99*
**IL-3**	11.50 ± 0.80	11.32 ± 1.24	15.05 ± 1.31	19.24 ± 2.29†
**IL-4**	3.51 ± 0.14	3.57 ± 0.05	59.88 ± 7.47	98.92 ± 25.54†
**IL-5**	11.87 ± 0.99	13.05 ± 0.54	26.95 ± 2.55†	33.30 ± 4.16†
**IL-6**	0.00	0.00	89.40 ± 3.40	108.37 ± 4.69‡
**IL-9**	0.00	0.00	968.40 ± 33.54	1030.62 ± 30.50
**IL-10**	24.35 ± 0.90	27.18 ± 0.67	373.72 ± 7.87*	462.43 ± 17.65*
**IL-12p40**	78.45 ± 17.87	49.06 ± 15.88	261.17 ± 31.22*	467.50 ± 44.09*
**IL-12p70**	96.28 ± 9.40	105.46 ± 5.66	510.79 ± 25.30*	691.97 ± 34.71*
**IL-13**	281.83 ± 15.57	307.55 ± 15.96	1720.70 ± 74.47*	2329.93 ± 150.05*
**IL-17A**	8.41 ± 0.35	9.41 ± 0.53	90.03 ± 3.20*	110.35 ± 6.73*
**EOTAXIN**	467.54 ± 24.21	538.83 ± 28.85	2255.97 ± 99.10*	2753.50 ± 87.87*
**G-CSF**	0.00	0.00	227.80 ± 28.41	126.43 ± 13.84‡
**GM-CSF**	123.00 ± 3.81	134.20 ± 5.47	184.52 ± 4.30†	186.91 ± 4.35†
**IFNγ**	28.26 ± 2.96	34.22 ± 3.71	65.86 ± 5.07	206.78 ± 80.14
**CXCL1**	32.33 ± 1.61	26.85 ± 1.56	346.10 ± 29.10*	268.39 ± 17.61*
**MCP-1**	85.90 ± 8.74	90.21 ± 5.75	851.93 ± 64.59†	1031.35 ± 99.39†
**MIP-1α**	36.29 ± 1.95	30.04 ± 2.20	197.01 ± 12.89†	178.24 ± 7.44†
**MIP-1β**	51.48 ± 2.39	50.75 ± 1.69	126.71 ± 7.14*	102.83 ± 6.50*
**CCL5**	45.90 ± 1.37	28.06 ± 1.72	680.98 ± 34.69*	516.05 ± 27.07*
**TNFα**	176.06 ± 5.68	194.75 ± 4.66	497.21 ± 10.94†	567.25 ± 37.35†

Values shown are mean ± SEM. Comparisons were made using an unpaired t-test (IL-6, IL-9, G-CSF) or an one-way ANOVA and Tukey’s post-test (all other analytes). Mice in control and CPI-203 groups received all ovalbumin challenges, but received phosphate buffered saline (PBS) retro-orbitally at time of cell transfer. *p < 0.05 when compared to all other groups, †p < 0.05 when compared to control and CPI-203 groups, and ‡p < 0.05 when compared to the Th17 group.

## References

[b1] Akinbami L. J., Moorman J. E. & Liu X. Asthma prevalence, health care use, and mortality: United States, 2005–2009. National health statistics reports, 1–14 (2011). 21355352

[b2] To T. . Global asthma prevalence in adults: findings from the cross-sectional world health survey. BMC Public Health 12, 204, doi: 10.1186/1471-2458-12-204 (2012). 22429515 PMC3353191

[b3] McKinley L. . TH17 cells mediate steroid-resistant airway inflammation and airway hyperresponsiveness in mice. J Immunol 181, 4089–4097, doi: 181/6/4089 [pii] (2008). 18768865 10.4049/jimmunol.181.6.4089 PMC3638757

[b4] Moore W. C. . Sputum neutrophil counts are associated with more severe asthma phenotypes using cluster analysis. J Allergy Clin Immunol 133, 1557–1563 e1555, doi: 10.1016/j.jaci.2013.10.011 (2014). 24332216 PMC4040309

[b5] Vazquez-Tello A., Halwani R., Hamid Q. & Al-Muhsen S. Glucocorticoid receptor-beta up-regulation and steroid resistance induction by IL-17 and IL-23 cytokine stimulation in peripheral mononuclear cells. J Clin Immunol 33, 466–478, doi: 10.1007/s10875-012-9828-3 (2013). 23160983

[b6] Nanzer A. M. . Enhanced production of IL-17A in patients with severe asthma is inhibited by 1alpha,25-dihydroxyvitamin D3 in a glucocorticoid-independent fashion. J Allergy Clin Immunol 132, 297–304 e293, doi: 10.1016/j.jaci.2013.03.037 (2013). 23683514

[b7] Manni M. L. . The complex relationship between inflammation and lung function in severe asthma. Mucosal immunology 7, 1186–1198, doi: 10.1038/mi.2014.8 (2014). 24549277 PMC4138304

[b8] Filippakopoulos P. & Knapp S. Targeting bromodomains: epigenetic readers of lysine acetylation. Nat Rev Drug Discov 13, 337–356, doi: 10.1038/nrd4286 (2014). 24751816

[b9] Delmore J. E. . BET bromodomain inhibition as a therapeutic strategy to target c-Myc. Cell 146, 904–917, doi: 10.1016/j.cell.2011.08.017 (2011). 21889194 PMC3187920

[b10] Dawson M. A. . Inhibition of BET recruitment to chromatin as an effective treatment for MLL-fusion leukaemia. Nature 478, 529–533, doi: 10.1038/nature10509 (2011). 21964340 PMC3679520

[b11] Zuber J. . RNAi screen identifies Brd4 as a therapeutic target in acute myeloid leukaemia. Nature 478, 524–528, doi: 10.1038/nature10334 (2011). 21814200 PMC3328300

[b12] Stuhlmiller T. J. . Inhibition of Lapatinib-Induced Kinome Reprogramming in ERBB2-Positive Breast Cancer by Targeting BET Family Bromodomains. Cell Rep 11, 390–404, doi: 10.1016/j.celrep.2015.03.037 (2015). 25865888 PMC4408261

[b13] Wyce A. . Inhibition of BET bromodomain proteins as a therapeutic approach in prostate cancer. Oncotarget 4, 2419–2429, doi: 10.18632/oncotarget.1572 (2013). 24293458 PMC3926837

[b14] Wyce A. . BET inhibition silences expression of MYCN and BCL2 and induces cytotoxicity in neuroblastoma tumor models. PloS one 8, e72967, doi: 10.1371/journal.pone.0072967 (2013). 24009722 PMC3751846

[b15] Nicodeme E. . Suppression of inflammation by a synthetic histone mimic. Nature 468, 1119–1123, doi: 10.1038/nature09589 (2010). 21068722 PMC5415086

[b16] Mele D. A. . BET bromodomain inhibition suppresses TH17-mediated pathology. J Exp Med 210, 2181–2190, doi: 10.1084/jem.20130376 (2013). 24101376 PMC3804955

[b17] Bandukwala H. S. . Selective inhibition of CD4+ T-cell cytokine production and autoimmunity by BET protein and c-Myc inhibitors. Proc Natl Acad Sci USA 109, 14532–14537, doi: 10.1073/pnas.1212264109 (2012). 22912406 PMC3437860

[b18] Belkina A. C., Nikolajczyk B. S. & Denis G. V. BET protein function is required for inflammation: Brd2 genetic disruption and BET inhibitor JQ1 impair mouse macrophage inflammatory responses. J Immunol 190, 3670–3678, doi: 10.4049/jimmunol.1202838 (2013). 23420887 PMC3608815

[b19] Zhang W. . Bromodomain-containing protein 4 (BRD4) regulates RNA polymerase II serine 2 phosphorylation in human CD4+T cells. J Biol Chem 287, 43137–43155, doi: 10.1074/jbc.M112.413047 (2012). 23086925 PMC3522308

[b20] Chen K. . Antiinflammatory effects of bromodomain and extraterminal domain inhibition in cystic fibrosis lung inflammation. JCI Insight 1, doi: 10.1172/jci.insight.87168 (2016). PMC4978187 27517095

[b21] Khan Y. M., Kirkham P., Barnes P. J. & Adcock I. M. Brd4 is essential for IL-1beta-induced inflammation in human airway epithelial cells. PloS one 9, e95051, doi: 10.1371/journal.pone.0095051 (2014). 24759736 PMC3997389

[b22] Perry M. M., Durham A. L., Austin P. J., Adcock I. M. & Chung K. F. BET bromodomains regulate transforming growth factor-beta-induced proliferation and cytokine release in asthmatic airway smooth muscle. J Biol Chem 290, 9111–9121, doi: 10.1074/jbc.M114.612671 (2015). 25697361 PMC4423696

[b23] Clifford R. L. . CXCL8 histone H3 acetylation is dysfunctional in airway smooth muscle in asthma: regulation by BET. American journal of physiology. Lung cellular and molecular physiology 308, L962–972, doi: 10.1152/ajplung.00021.2015 (2015). 25713319 PMC4421784

[b24] Tang X. . Assessment of Brd4 inhibition in idiopathic pulmonary fibrosis lung fibroblasts and *in vivo* models of lung fibrosis. The American journal of pathology 183, 470–479, doi: 10.1016/j.ajpath.2013.04.020 (2013). 23759512

[b25] Stefanowicz D. . Elevated H3K18 acetylation in airway epithelial cells of asthmatic subjects. Respir Res 16, 95, doi: 10.1186/s12931-015-0254-y (2015). 26243279 PMC4531814

[b26] Matthews J. G., Ito K., Barnes P. J. & Adcock I. M. Defective glucocorticoid receptor nuclear translocation and altered histone acetylation patterns in glucocorticoid-resistant patients. J Allergy Clin Immunol 113, 1100–1108, doi: 10.1016/j.jaci.2004.03.018 (2004). 15208591

[b27] Ito K. . Expression and activity of histone deacetylases in human asthmatic airways. Am J Respir Crit Care Med 166, 392–396, doi: 10.1164/rccm.2110060 (2002). 12153977

[b28] Butler C. A. . Glucocorticoid receptor beta and histone deacetylase 1 and 2 expression in the airways of severe asthma. Thorax 67, 392–398, doi: 10.1136/thoraxjnl-2011-200760 (2012). 22156779

[b29] Bergeron C. . Increased glucocorticoid receptor-beta expression, but not decreased histone deacetylase 2, in severe asthma. J Allergy Clin Immunol 117, 703–705, doi: 10.1016/j.jaci.2005.12.1344 (2006). 16522474

[b30] Alcorn J. F. . Transforming growth factor-beta1 suppresses airway hyperresponsiveness in allergic airway disease. Am J Respir Crit Care Med 176, 974–982, doi: 10.1164/rccm.200702-334OC (2007). 17761617 PMC2078678

[b31] Manni M. L. . Molecular Mechanisms of Airway Hyperresponsiveness in a Murine Model of Steroid-Resistant Airway Inflammation. J Immunol, doi: 10.4049/jimmunol.1501531 (2016). PMC4724491 26729801

[b32] Evans C. M., Kim K., Tuvim M. J. & Dickey B. F. Mucus hypersecretion in asthma: causes and effects. Curr Opin Pulm Med 15, 4–11, doi: 10.1097/MCP.0b013e32831da8d3 (2009). 19077699 PMC2709596

[b33] Long A. J. . Gob-5 contributes to goblet cell hyperplasia and modulates pulmonary tissue inflammation. American journal of respiratory cell and molecular biology 35, 357–365, doi: 10.1165/rcmb.2005-0451OC (2006). 16645179

[b34] Kudo M. . IL-17A produced by alphabeta T cells drives airway hyper-responsiveness in mice and enhances mouse and human airway smooth muscle contraction. Nat Med 18, 547–554, doi: 10.1038/nm.2684 (2012). 22388091 PMC3321096

[b35] Chesne J. . Prime role of IL-17A in neutrophilia and airway smooth muscle contraction in a house dust mite-induced allergic asthma model. J Allergy Clin Immunol 135, 1643–1643 e1643, doi: 10.1016/j.jaci.2014.12.1872 (2015). 25649077

[b36] Willis C. R. . IL-17RA Signaling in Airway Inflammation and Bronchial Hyperreactivity in Allergic Asthma. Am J Respir Cell Mol Biol 53, 810–821, doi: 10.1165/rcmb.2015-0038OC (2015). 25919006

[b37] Moros A. . Synergistic antitumor activity of lenalidomide with the BET bromodomain inhibitor CPI203 in bortezomib-resistant mantle cell lymphoma. Leukemia 28, 2049–2059, doi: 10.1038/leu.2014.106 (2014). 24721791

[b38] Wong C. . The bromodomain and extra-terminal inhibitor CPI203 enhances the antiproliferative effects of rapamycin on human neuroendocrine tumors. Cell Death Dis 5, e1450, doi: 10.1038/cddis.2014.396 (2014). 25299775 PMC4237236

[b39] King B. . The ubiquitin ligase FBXW7 modulates leukemia-initiating cell activity by regulating MYC stability. Cell 153, 1552–1566, doi: 10.1016/j.cell.2013.05.041 (2013). 23791182 PMC4146439

[b40] Chaidos A., Caputo V. & Karadimitris A. Inhibition of bromodomain and extra-terminal proteins (BET) as a potential therapeutic approach in haematological malignancies: emerging preclinical and clinical evidence. Ther Adv Hematol 6, 128–141, doi: 10.1177/2040620715576662 (2015). 26137204 PMC4480520

